# Epidemiological and Molecular Approaches for a Fatal Feline Panleukopenia Virus Infection of Captive Siberian Tigers (*Panthera tigris altaica*) in the Republic of Korea

**DOI:** 10.3390/ani13182991

**Published:** 2023-09-21

**Authors:** Yong-Gu Yeo, Hye-Ryung Kim, Jonghyun Park, Jong-Min Kim, Yeun-Kyung Shin, Kyoung-Ki Lee, Oh-Kyu Kwon, Hye-Young Jeoung, Hae-Eun Kang, Bok-Kyung Ku, Seung-Chun Park, Oh-Deog Kwon, Choi-Kyu Park

**Affiliations:** 1 College of Veterinary Medicine, Kyungpook National University, Daegu 41566, Republic of Korea; withyonggu@seoul.go.kr (Y.-G.Y.); gpfuddl25@knu.ac.kr (H.-R.K.); kjm51062@knu.ac.kr (J.-M.K.); 2 Seoul Zoo, Gwacheon 13829, Republic of Korea; 3 Laboratory of Veterinary Pharmacokinetics and Pharmacodynamics, College of Veterinary Medicine and Cardiovascular Research Institute, Kyungpook National University, Daegu 41566, Republic of Korea; parkjh@knu.ac.kr (J.P.); parksch@knu.ac.kr (S.-C.P.); 4 Animal and Plant Quarantine Agency, Gimcheon 39660, Republic of Korea; shinyk2009@korea.kr (Y.-K.S.); naturelkk@korea.kr (K.-K.L.); kwonok59@korea.kr (O.-K.K.); jhy98@korea.kr (H.-Y.J.); kanghe@korea.kr (H.-E.K.); kubk@korea.kr (B.-K.K.)

**Keywords:** feline panleukopenia virus, captive Siberian tiger, epidemiology, molecular analysis, Republic of Korea

## Abstract

**Simple Summary:**

A fatal feline panleukopenia virus (FPV) infection occurred in endangered Siberian tigers living in a zoo in the Republic of Korea. Genetic and phylogenetic analyses revealed that the infection was caused by the cross-species transmission of cat-derived FPV prevalent in Korea. The vaccinated tigers were infected with FPV most likely due to insufficient protective immunity or vaccine failure triggered by maternal-derived antibody interference. Therefore, improved biosecurity and vaccination guidelines are required to save the lives of endangered wild carnivores from fatal FPV infection. The data presented in this study will help us expand our knowledge of the molecular epidemiology of FPV in Korea and establish an effective control plan to prevent future FPV infections in captive wild carnivores.

**Abstract:**

Feline panleukopenia virus (FPV), a member of the species *Protoparvovirus carnivoran1*, is one of the most fatal pathogens of domestic and wild carnivores. The virus endemically infects domestic carnivores worldwide and its cross-species transmission threatens endangered wild carnivores, including Siberian tigers. In this study, a fatal FPV infection in endangered Siberian tigers was investigated to trace the origin of the virus and elucidate the reason behind FPV’s infection of the vaccinated tigers. Our genetic characterization and phylogenetic analysis revealed that the virus detected in the infected tigers, designated as the KTPV-2305 strain, was closely related to FPV strains circulating in Korean cats, suggesting that it might have been transmitted from stray cats wandering around the zoo. Compared with the prototype FPV reference strains, the KTPV-2305 strain carried three distinct amino acid (aa) mutations in the VP2 protein sequence (I101T, I232V, and L562V) in this study. These three mutations are commonly found in most global FPV strains, including Korean strains, indicating that these mutations are common evolutionary characteristics of currently circulating global FPVs. The reason why the vaccinated tigers were infected with FPV was most likely the insufficient protective immunity of the affected tigress or vaccine failure triggered by the interference of maternal-derived antibodies in the affected tiger cubs. These findings suggest that improved vaccination guidelines are urgently needed to save the lives of wild carnivores from this fatal virus.

## 1. Introduction

Feline panleukopenia, caused by feline panleukopenia virus (FPV), is one of the most serious diseases in domestic cats, characterized by anorexia, vomiting, diarrhea, leukopenia, and high mortality in kittens [1]. FPV is currently classified into a single species, *Protoparvovirus carnivoran1*, in the genus *Protoparvovirus* of the family *Parvoviridae*, along with closely related canine parvovirus 2 (CPV-2) [2]. The viruses are non-enveloped icosahedral viruses with a single-stranded DNA genome comprising two open reading frames encoding two nonstructural proteins (NS1 and NS2) and two structural proteins (VP1 and VP2) each. VP2 sequencing and phylogenetic analyses have been widely used in molecular epidemiological studies of parvovirus infections of domestic and wild carnivores because they contain key epitopes influencing viral pathogenicity, immune response, and host range [3,4].

As viral DNA synthesis relies on cellular DNA polymerases, FPV replicates in rapidly dividing cells in the intestines, lymphoid tissue, and bone marrow of infected cats, which exhibit clinical presentations of viral infection, such as vomiting, diarrhea, dehydration, lymphopenia, and panleukopenia. The outcome of FPV infection in cats ranges from subclinical to fatal infection with high mortality depending on the cat’s age, immune status, and concurrent infections [5]. Diseased cats shed viruses in a high concentration, which quickly accumulate in affected premises. FPV is highly resistant to physical factors and chemical substances; therefore, it retains its infectivity in an environment for weeks or even months, which makes it easy for this virus to spread directly through animal-to-animal contact and indirectly through various contaminated fomites [1].

Due to the ubiquitous distribution of the virus and critical outcomes of the infection, FPV vaccination is highly recommended as a feline core vaccine that all domestic cats should be routinely inoculated with [6]. Both modified live virus (MLV) and killed vaccines are considered to provide solid immunity against FPV infection; however, MLV vaccines are preferred over killed vaccines due to the rapid onset of immunity and better resistance to maternal-derived antibodies (MDAs). Cats successfully immunized with FPV vaccines maintain protective immunity against FPV for many years [1,6,7]. Currently, FPV infections are rarely observed in regularly vaccinated pet cats but are still problematic in stray cats or cats in animal shelters without FPV vaccines administered.

Apart from domestic cats, wild felids are also susceptible to FPV infection, and the clinical outcomes are often fatal, threatening the survival of endangered wild animals such as Siberian tigers [4,8]. The Siberian tiger (*Panthera tigris altaica*), also known as the Amur tiger, is listed as an endangered species by the International Union for the Conservation of Nature. Currently, about 500 Siberian tigers remain in the wild, but many are being bred in zoos around the world [9,10]. Although captive tigers are bred in comfortable circumstances with ample food and without natural enemies, they are more vulnerable to FPV infections as they share habitats with domestic cats that are potential reservoirs of FPV. Indeed, fatal FPV infections have been reported in captive Siberian tigers throughout the world, including Portugal [8], Germany [4], China [10,11], and Korea [12]. To protect the endangered species from fatal FPV infections, more extensive epidemiological studies are required to explore the sources of infection and transmission routes of FPV to establish more advanced control strategies for FPV as well as other infectious pathogens. However, so far, there have been few epidemiological studies on FPV infection in captive Siberian tigers.

In May 2023, panleukopenia-suspected clinical signs were observed in captive Siberian tigers at a Korean zoo. The tigers had been vaccinated with commercial FPV MLV vaccines, and one of the infected tigers died after showing severe clinical signs. After the outbreak, an extensive epidemiological investigation was conducted, but the origin and transmission route of the virus have not yet been elucidated. Therefore, the purpose of this study was to identify the origin of this FPV infection through a genetic characterization of the virus and to explore the scientific reason why these tigers were not protected from FPV infection despite being vaccinated with multiple doses of commercial vaccines before the FPV outbreak. The findings of this study expand our knowledge regarding the molecular epidemiology of FPV infection in Korea and establish improved control plans to protect captive wildlife from fatal viral infections, which will contribute to conserving endangered wild animal species.

## 2. Materials and Methods

### 2.1. Clinical and Epidemiological Data Collection

Clinical and epidemiological data of the Siberian tigers were collected to understand the epidemiology of the disease outbreak at Seoul Zoo in Gwacheon city, Gyeonggi-do province, the Republic of Korea. The basic epidemiological information collected included housing practices, vaccination history, clinical manifestation and length of the infection, contacts with other Siberian tigers in the same facility or nearby facilities, and the presence of stray cats roaming around the tigers’ facilities.

### 2.2. Sample Collection

Fecal samples were collected from five Siberian tigers, including three diseased tigers (two cubs and their mother) and other two Siberian tigers without clinical signs housed in a nearby facility. As the fecal samples to be used in this study were collected by veterinarians at Seoul Zoo without any animal contact, ethical approval from our institute regarding the animals was not required. Prior to our analysis, fecal samples were screened for parvovirus antigens using a commercial immunochromatography assay kit (Anigen Rapid FPV Ag Test Kit, BioNote, Inc., Hwaseong, Republic of Korea) according to the manufacturer’s protocol. Subsequently, each fecal sample was immersed in 1 mL of phosphate-buffered saline (0.15 M, pH 7.2) and then centrifuged at 3000× *g* for 3 min. Then, total nucleic acids were extracted from 200 µL of each supernatant using a TANBead nucleic acid extraction kit equipped with a fully automated magnetic bead operating platform (Taiwan Advanced Nanotech Inc., Taoyuan, Taiwan). The extract was eluted with 100 µL elution buffer according to the manufacturer’s instructions and stored at −80 °C until use. Whole blood samples were collected from each affected tiger via venipuncture of the jugular vein and transferred to a BD Vacutainer^®^ blood collection tube containing anticoagulant (Becton, Dickinson and Company, Franklin Lakes, NJ, USA). White blood cells (WBCs) were counted using an automatic hematology analyzer (IDEXX ProCyte DM^TM^, IDEXX Laboratories Inc., Westbrook, ME, USA) according to the operator’s guide.

### 2.3. Molecular Assays for Screening of Viral Pathogens

Molecular analyses were performed on all samples to screen for FPV, feline coronavirus (FCoV), feline calicivirus (FCV), canine distemper virus (CDV), and feline leukemia virus (FeLV) using THUNDERBIRD™ Probe One-step quantitative reverse-transcription polymerase chain reaction (qRT-PCR) kit (TOYOBO, Osaka, Japan) according to the manufacturer’s instructions. The primers and probes used in this study are shown in Table 1. For FPV, qPCR assay without a reverse-transcription step was performed using primers and probe set, amplifying genes encoding the structural protein VP2 of both FPV and CPV [13]. For FCoV, FCV, CDV, and FeLV, qRT-PCR assays were performed using each viral gene-specific primer and probe as previously described [14,15,16,17].

### 2.4. VP2 Gene Sequencing

To genetically characterize FPVs detected from Siberian tigers in this study, complete VP2 gene sequences were obtained from two FPV-positive samples with low Ct values. To obtain complete VP2 gene fragment, PCR was performed with a pair of primers (forward primer, 5′-GAGACAATCTTGCACCAATGAG-3′ and reverse primer, 5′-AGTATATTAATATAATTTTCTAGGTGCTA-3′) using Takara Ex Taq (Takara Korea Biomedical Inc., Seoul, Korea) as described by An et al. (2011) [18]. A PCR product was generated containing the complete VP2 gene (1755 bp), which encodes 584 amino acids (aa). The amplified PCR products were purified using a commercial kit (GeneAll Expin™ Combo GP 200 miniprep kit, GeneAll, Seoul, Republic of Korea) and sequenced using Sanger’s method in duplicate via a commercial company (BIONICS, Daejeon, Republic of Korea).

### 2.5. Sequence and Phylogenetic Analyses

The VP2 gene sequences of 595 global FPV strains derived from various hosts were retrieved from the GenBank database (www.ncbi.nlm.nih.gov/genebank (accessed on 30 May 2023)). Eighteen Australian FPV sequences (GenBank accession numbers, MZ742163–MZ742180) were excluded from this study because their start codons were not definite. The VP2 nucleotide (nt) and aa sequence of the KTPV-2305 strain obtained in this study were aligned with corresponding sequences of the selected 574 global FPV strains, including three FPV reference strains, and four reference CPV-2 strains using MAFFT multiple sequence alignment software (v7.490) [19]. Based on the MAFFT alignment, pairwise nt and aa sequence identities were determined by Geneious Prime (https://www.geneious.com, accessed on 1 June 2023). For the phylogenetic analysis, the IQ-TREE v2.0 software package (http://www.iqtree.org, accessed on 6 June 2023) was used in this study [20]. The best-fit substitution model (GTR + F + I + G4) was selected by ModelFinder [21], and a maximum likelihood phylogenetic tree was constructed using ultrafast bootstrap analysis with 1000 replicates [22]. The phylogenetic tree was visualized using the iTOL phylogenetic tree viewer [23].

## 3. Results

### 3.1. Clinical and Epidemiological Features of the Infected Tigers

**Figure 1:**
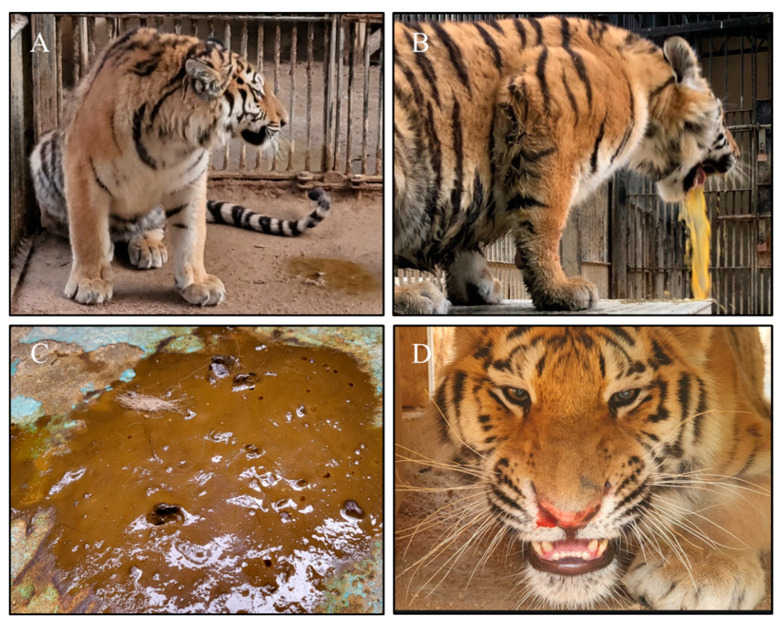
Clinical signs observed in Siberian tigers (*Panthera tigris altaica*). Depression (**A**), vomiting (**B**), diarrhea (**C**), and epistaxis (**D**) were commonly observed in affected tiger cubs.

Prior to the disease outbreak, 13 Siberian tigers were housed alone or in pairs in separate breeding houses with their own playground at Seoul Zoo located in Gwacheon city, Gyeonggi province, the Republic of Korea. As an exception, four tigers including three one-year-old sibling cubs (named Haerang, Sarang, and Parang, respectively) and a 13-year-old mother tigress (named Penza) were housed in the same breeding house. Starting from 2 May 2023, the four Siberian tigers housed in the same breeding house became sick, showing clinical signs of depression, anorexia, vomiting, diarrhea, nasal secretions, epistaxis, and/or dyspnea (Figure 1). Using a commercial immunochromatography assay kit, the FPV antigen was identified in the fecal samples of all four sick tigers. The hematological examination results showed that the WBC values (×10^3^/μL) of the affected tiger cubs were 4.89 (Haerang), 3.08 (Sarang), and 0.66 (Parang) (Table 2), which were much lower than the mean value (10.99) reported in a previous study on Siberian tigers [24]. Symptoms were more severe in the three one-year-old siblings than in the mother, who recovered completely from the disease after showing only transient, mild anorexia for two days. One cub (Parang) died on the third day after showing the most severe clinical signs and leukopenia; however, the remaining two cubs recovered to a healthy state after two weeks of extensive supportive care and treatment. The one-year-old sibling cubs had been administrated three doses of a commercial feline vaccine containing the attenuated FPV Philips Roxane strain (Nobivac^®^ Feline 1-HCPCh + FeLV, Intervet Inc., Merck Animal Health, Rahway, NJ, USA) at 7, 12, and 16 weeks of age. The mother tigress had been annually vaccinated with the FPV vaccine since 2011 and the last dose had been administrated in June 2022. Based on the vaccination schedule of the animals in the zoo, the affected siblings were supposed to receive a booster vaccination in May 2023, which was not executed due to the unexpected disease outbreak. However, according to the zoo’s officials, stray cats were often seen roaming around the facilities where the Siberian tigers were housed; thus, there was a potential risk of the virus spreading from stray cats to the tigers through indirect contact or fomites.

### 3.2. Detection of Viral Pathogens

**Figure 2:**
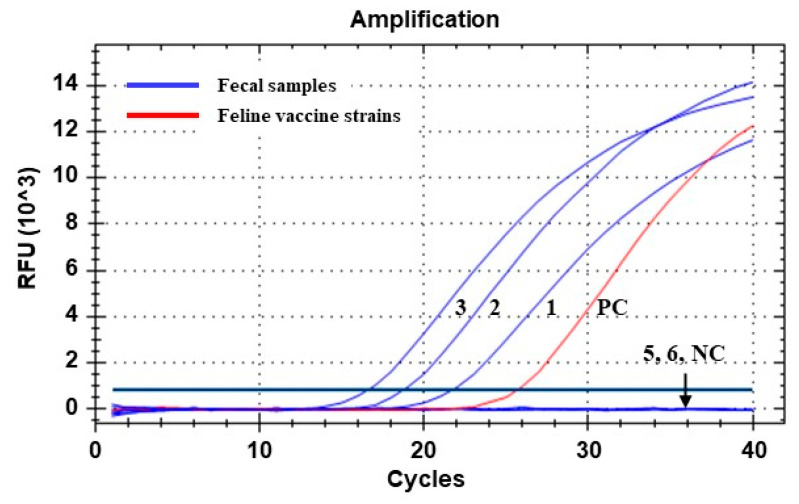
Amplification curves generated via qPCR assay for detecting parvoviral DNA from fecal samples of Siberian tigers (*Panthera tigris altaica*). Lines 1 to 6 correspond to samples 1, 2, 3, 5, and 6 in Table 2, respectively. Line PC, positive control (DNA sample extracted from a commercial feline vaccine containing FPV Philips Roxane strain). Line NC, negative control (nuclease-free water). Ct values of the positive samples (lines 1, 2, and 3) were determined as 21.62, 18.65, and 16.49, respectively.

Out of the five fecal samples collected from the Siberian tigers, three samples were identified as positive for FPV/CPV via the qPCR assay. All three of the FPV/CPV-positive samples were from clinically ill tigers housed in the same facility, whereas the other two FPV/CPV-negative samples were from clinically healthy tigers housed in a nearby facility (Table 2). Moreover, the fecal samples were tested for FCoV, FCV, CDV, and FeLV via qRT-PCR assays and were negative for all four of the tested viruses (Table 2). These results demonstrated that the three FPV/CPV-positive tigers were infected with parvovirus only. The Ct values generated by the qPCR assay for the fecal samples collected from the two severely affected tiger cubs were 18.65 and 16.49, respectively, which were lower than that of the sample collected from the mother tiger with mild clinical signs (Table 2 and Figure 2). Two DNA samples with low Ct values were used for a subsequent genetic analysis in this study.

### 3.3. Analysis of VP2 Gene Sequence of the KTPV-2305 Strain

To characterize the FPV detected from diseased tigers, complete VP2 gene sequences were obtained from two positive fecal samples of the affected Siberian tigers. The alignment of the two sequences showed that they were 100% identical to each other, and thereafter, a further analysis was performed with a sequence of the KTPV-2305 strain (GenBank accession number: OR365078). The complete VP2 gene sequence of the KTPV-2305 strain was compared to corresponding sequences of the FPV and CPV-2 reference strains as well as 33 Korean FPV strains available in GenBank (Table 3). The VP2 nt or aa sequence of the KTPV-2305 strain shared 98.6–98.9% or 97.8–98.1% homology with the CPV-2 reference strains, 99.5% or 99.5–99.7% homology with the FPV reference strains, and 98.2–100% or 95.9–100% homology with the previously reported 33 Korean FPV strains, respectively (Table 3). Among the 33 Korean FPV strains, the K4 strain detected from a Korean domestic cat in 2008 was the closest to the KTPV-2305 strain, with 100% nt and aa homology. Additionally, eight other strains that were detected from domestic cats in 2008 (K3), 2017 (17D01 and 17D02), and 2019 (19D01, 19D02, 19D03, 19D04, and 19SP_CK-8) were also closest to the KTPV-2305 strain, with 100% aa homology. These results suggested that the KTPV-2305 strain might be derived from currently circulating FPVs in the Korean cat population.

To further analyze the mutation of the KTPV-2305 strain, the aa sequence of VP2 was compared with that of the reference CPV-2 and FPV, as well as Korean FPV strains derived from cats (Table 4). The six key aa residues of the VP2 protein that were related to the antigenicity and host specificity of the virus were determined as 80 K, 93 K, 103 V, 323 D, 564 S, and 568 G in the KTPV-2305 strain from the Siberian tigers in this study, which were consistent with the reference FPV strains. However, three aa mutations were identified at the sites 101 (Ile to Thr), 232 (Ile to Val), and 562 (Leu to Val) in the VP2 sequence of the KTPV-2305 strain relative to the VP2 sequences of the reference FPV strains, and these mutations were found to be common in the FPV strains derived from Korean cats. Out of the 34 Korean FPVs, including the KTPV-2305 strain analyzed in this study, 34 (100.0%), 32 (94.1%), and 33 (97.1%) showed I101T, I232V, and L562V mutations, respectively. To enrich our understanding of the occurrence of these three mutations, 578 global FPV strains, including three reference strains, were retrieved from the GenBank database, and the aa residues at the 101, 232, and 562 positions of the VP2 protein of each strain were determined (Table S1). The results showed that the I101T, I232V, and L562V mutations were observed in 97.8% (565/578), 91.3% (528/578), and 96.2% (556/578) of global FPV strains, respectively.

### 3.4. Phylogenetic Analysis Based on VP2 Sequences of FPVs

**Figure 3:**
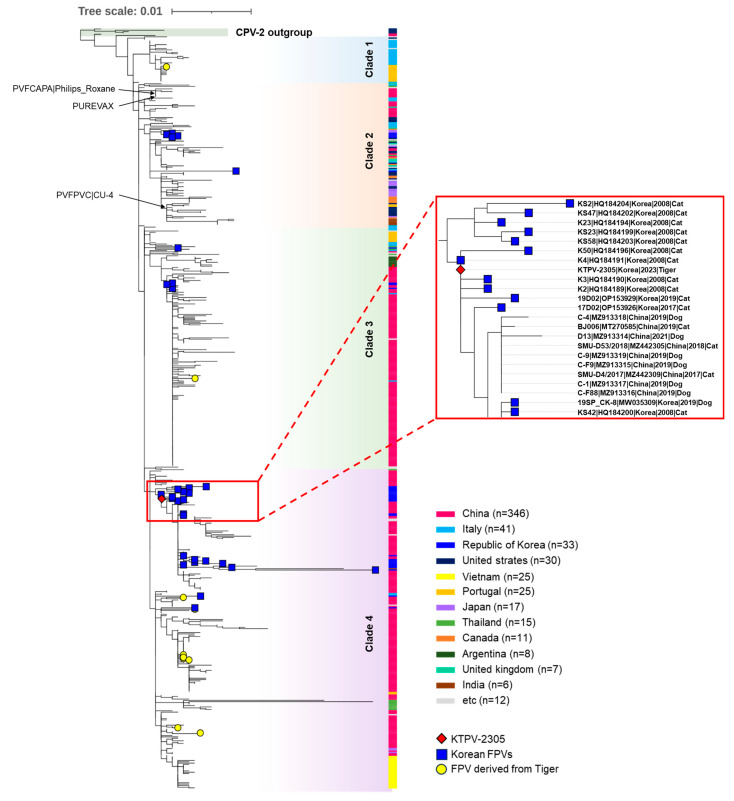
A phylogenetic tree was constructed using the maximum likelihood method from complete VP2 gene nucleotide sequences of FPV strains assessed using 1000 bootstrap replications. The phylogenetic tree was visualized using the iTOL phylogenetic tree viewer. The red diamond represents the KTPV-2305 strain detected from the Siberian tiger (*Panthera tigris altaica*) in this study. Blue squares indicate previously reported FPV strains from Korean cats. Yellow circles indicate FPV strains derived from tigers worldwide. Black arrows indicate FPV vaccine strains. Others are global FCV strains and CPV reference strains labeled with their strain name, GenBank accession number, country, year, and animal host. Scale bars indicate nucleotide substitutions per site.

For the phylogenetic analysis of the KTPV-2305 strain, a phylogenetic tree was constructed using the VP2 sequences of 578 global FPV strains and four CPV-2 reference strains retrieved from the GenBank database (Table S1). In the phylogenetic tree, global FPVs were classified into four clades (clade 1–4) distinct from the CPV-2 outgroup (Figure 3). Although no clear temporal or geographical grouping pattern was observed in the constructed phylogenetic tree, the 33 previously reported Korean FPVs were distributed among the groups as follows: clade 2 (*n* = 5), clade 3 (*n* = 4), and clade 4 (*n* = 24). The KTPV-2305 strain detected from the Siberian tigers in this study was grouped into clade 4 and was closely related to previously reported Korean FPV strains detected in domestic or stray cats in 2008, 2017, and 2019 (Figure 3), indicating the possible transmission of FPVs between the Siberian tigers and Korean cats.

## 4. Discussion

FPV, a member of the species *Protoparvovirus carnivoran1*, is one of the fatal viral pathogens that can be cross-transmitted between domestic and wild carnivores, and FPV infection has been reported in various wild carnivores including Siberian tigers, threatening their survival [4,8,10,11,12]. In early May 2023, some Siberian tigers housed at Seoul Zoo showed clinical signs, such as depression, anorexia, vomiting, diarrhea, and epistaxis, and were suspected to be infected with a fatal enteric disease (Figure 1). These sick tigers were confirmed to be infected with parvovirus via the previously described qPCR assay (Table 2 and Figure 2). Given that the qPCR assay used in this study was developed with the primers and probe set targeting the VP2 gene of all members of the *Protoparvovirus carnivoran1* species including FPV, CPV-2, and its variants [13], further analyses were necessary to specify whether the detected virus was FPV or CPV-2. Indeed, previous reports have shown that tigers can be infected with genetically different parvoviruses, including FPV [8,10,11,25] and CPV-2a [4,26]. Therefore, to further characterize the detected KTPV-2305 strain, a complete VP2 gene sequence (GenBank accession number: OR365078) was obtained and analyzed in this study. Although most of the recently published FCV sequences were derived from China (Figure 3 and Table S1), this was not expected to adversely affect the phylogenetic analysis; on the contrary, it could provide meaningful epidemiological information due to the geographical proximity between China and Korea in this study. The sequence and phylogenetic analyses based on the VP2 gene revealed that the KTPV-2305 strain was classified into clade 4 along with majority of previously reported Korean FPV strains (24/33, 72.7%) (Table 3 and Figure 3) and was most closely related to some Korean FPV strains reported in 2008, 2017, and 2019 [18,27], suggesting that the FPV detected from the captive Siberian tigers in this study might have originated from Korean cats probably due to cross-species transmission between the tigers and stray cats. These findings are predictable as previous studies have raised the possibility of the cross-species transmission of parvovirus via indirect contact with contaminated fomites of domestic cats and dogs [4,8,10,11,25,28].

In the Republic of Korea, three fatal parvoviral infection cases in captive tigers have been reported, and these parvoviral infections were diagnosed via virus isolation, a pathological examination, antigen detection, and/or viral gene amplification. However, the origin of the virus could not be determined in these three Korean studies because a genetic analysis of the infecting virus was not conducted [12,29,30]. In this study, we first characterized the FPV infection in the captive Siberian tigers and determined the infection source of the virus through molecular approaches in the Republic of Korea. In a recent study conducted in Gyeonggi-do province, Korea, where Seoul Zoo is located, 76.5% (156/204) of stray cats were infected with FPV, and the seroprevalence of FCV reached 92.5% (185/200) [31]. The extremely high prevalence of FPV in stray cats sharing habitats with the captive tigers strongly supports the idea that infected stray cats played an important role in the cross-species transmission of FPV to the captive tigers at Seoul Zoo. However, FPV infections in stray cats wandering near Seoul Zoo were not confirmed in this study. Therefore, further studies are required to investigate the occurrence of FCV in stray cats wandering around the zoo and to establish control measures for preventing the potential transmission of the pathogens from stray cats to captive animals.

The VP2 protein sequence analysis revealed that the KTPV-2305 strain carried FPV-specific aa residues that were consistent with those of reference FPV strains, but the KTPV-2305 strain showed three aa mutations (I101T, I232V, and L562V), which were distinct from those of reference FPV strains but were identified in almost all FPV strains throughout the world, including in Korea (Table 4 and Table S1). Although FPV is known to be genetically stable, several mutations in the VP2 sequence have been identified in FPVs from different hosts, such as the A91S mutation of cat-derived FPVs [32], the K93N mutation of dog-derived FPVs [33], the I101T mutation of tiger- and dog-derived FPVs [10,34,35], the I232V mutation of cat-derived FPVs [32], the F299E mutation of captive giant-panda-derived FPVs [36], and the V300A mutation of mink-derived FPVs [37].

According to previous studies, the I101T mutation of FPV strains was first identified in a tiger parvovirus (CHJL-Siberian Tiger-01/2017 strain) isolated from a captive Siberian tiger in China [10] and was then further identified in other Siberian tigers in China [11] as well as dogs in Vietnam [35] and Italy [34]. The authors suggested that the I101T mutation was potentially unique in tiger- or dog-associated FPV strains. However, unlike the previous reports, the I101T mutation was commonly observed in all FPV strains detected from Korean cats during 2007 and 2019 (Table 4). Moreover, a further analysis of 578 global FPV strains showed that the I101T mutation was commonly observed in 565 (97.8%) of the FPV strains derived from various hosts in this study (Table S1), indicating that the I101T mutation is common in almost all FPV strains but is not uniquely observed in FPV strains from Siberian tigers or Vietnamese and Italian dogs [10,11,34,35]. For the I232V mutation, a previous Chinese study reported that all 55 FPV sequences obtained from hospitalized cats in Beijing carried the I232V mutation and suggested that it might be a novel pattern of VP2 genetic evolution in FPV strains in Beijing [32]. The I232V mutation was identified in FPV strains from Korea (about 94%) and other countries (about 91%) analyzed in this study (Table 4 and Table S1). The L562V mutation was also identified in previously published FPV strains from Korea (approximately 97%) and other countries (approximately 96%) (Table 4 and Table S1). These results indicated that the KTPV-2305 strain detected in the Siberian tiger shared a common evolutionary characteristic with currently circulating global FPVs including Korean FPVs. The substitution of aa residues in VP2 protein might lead to changes in the viral surface structure, which influence the host range and pathogenicity of both FPV and CPV-2 [38,39,40]. The I101T mutation is suggested to be related to the determination of host range [35,41], but the functional consequences of the I232V and L562V mutations remain unknown. Therefore, further studies are needed to elucidate the potential impacts of these three aa mutations of the VP2 protein on the host range and pathogenesis of FPV infections.

As FPV infection is a fatal disease in domestic cats, several FPV vaccines are commercially available and widely used as core vaccines according to the guidelines for feline vaccination recommended by the World Small Animal Veterinary Association (WSAVA) [6,42]. However, there are no approved vaccines and recommended vaccination guidelines for non-domestic felids including wild felids in zoos. Therefore, commercial vaccines approved for domestic cats have been generally used for non-domestic felids in accordance with the vaccination guideline for domestic cats [43]. In this study, four tigers were infected with FPV and one of them died, despite receiving multiple doses of commercial FPV vaccines prior to the infection. The reason for this is unknown, but exploring the reason may help prevent further FPV infections in captive wild carnivores. The 13-year-old mother tigress recovered from the FPV infection after showing only transient anorexia for two days, which indicated that the tigress had enough protective immunity to prevent clinical aggravation resulting from FPV infection, unlike the affected tiger cubs, which showed severe and fatal clinical signs. The tigress was believed to have had a solid immunity due to prolonged annual revaccination. Therefore, possible reasons for the tigress showing mild clinical signs might be from overwhelming exposure to the virus, immune suppression associated with unknown stress, or concurrent infection with other diseases, particularly immunosuppressive diseases [42,44]. However, no co-infections with common viral pathogens (FCoV, FCV, CDV, and FeLV) were detected in the fecal samples of the affected tigers including the tigress (Table 2). Therefore, the vulnerability of the tigress against FPV infection was presumed to be due to overwhelming exposure to the virus or unknown stress. The infected 1-year-old Siberian tiger cubs had received three doses of the FPV vaccine at 7, 12, and 16 weeks of age according to the previously published vaccination guidelines for domestic cats and wild carnivores [6,43]. However, despite being vaccinated according to the recommended guidelines, all three tiger cubs were infected with FPV and exhibited FPV-specific clinical signs. One of them even died, which suggested that their vaccine-induced immunity was not sufficient to fully protect them against FPV at the time of infection. To understand the reason why the tiger cubs were not protected from FPV infection despite vaccination, we concentrated on the host-related vaccine failure induced by MDA, which is well documented in young animals and has been recognized as one of the most common reasons for vaccine failure in cats and dogs [45,46,47,48]. According to this hypothesis, kittens that receive high levels of MDA may fail to respond to a final dose of the FPV vaccine at 16 weeks of age. Therefore, the WASVA recently published an updated feline vaccination guideline recommending the revaccination of the FPV vaccine at 6 months of age instead of 1 year of age [42]. As the affected tiger cubs were not revaccinated, even though at least 9 months had elapsed since their last vaccination at 16 weeks of age, it is very likely that they were infected with FPV because their vaccinal immunity had fallen below the protective level at the time of the viral infection. To fully prove the vaccine failure described above, serological evidence is required to assess the presence of MDA in the affected tiger cubs. It was not possible to discover serological evidence in this study because no serum samples from the tiger cubs were collected prior to the outbreak. Therefore, further studies are necessary to characterize the immune response of Siberian tigers to ensure the effectiveness of the vaccination protocols against leading infectious diseases. More importantly, an improved vaccination guideline for FPV is urgently needed to overcome vaccine failure due to MDA interference in order to save Siberian tiger cubs residing in zoos from fatal FPV infection.

## 5. Conclusions

In this study, a fatal FPV infection in endangered Siberian tigers was investigated to track the origin of the virus and elucidate the reason behind the FPV infection of the tigers despite them being vaccinated against the virus. Genetic characterization and a phylogenetic analysis revealed that the virus detected in the diseased tigers was closely related to FPV strains prevalent in Korean cats, suggesting that the virus was transmitted from the stray cats roaming around the zoo to the tigers. The tigers were infected with FPV despite receiving multiple doses of a commercial vaccine most likely due to insufficient protective immunity or vaccine failure triggered by MDA interference, indicating that more effective vaccination guidelines are needed to save wild carnivores from fatal viral pathogens. The findings of this study will help expand our knowledge of the molecular epidemiology of FPV in Korea and establish control measures to prevent future FPV infections in captive wild carnivores.

## Figures and Tables

**Table 1:** Primers and probes for screening target genes in viral pathogens in this study.

Pathogen	Target Gene	Primers and Probe (5′-3′) ^a^	Amplicon Size (bp)	References
FPV/CPV	VP2	F: CGGGGGTGGTGGTGGTT R: GCTTGAGTTTGCTGTGATTTCC P: FAM-CTGGGGGTGTGGGGATTTCTACG-BHQ1	112	Cao et al. (2022) [13]
FCoV	7b	F: GATTTGATTTGGCAATGCTAGATTT R: AACAATCACTAGATCCAGACGTTAGCT P: FAM-TCCATTGTTGGCTCGTCATAGCGGA-BHQ1	102	Gut et al. (1999) [15]
FCV	P30	F: GCCAATCAACATGTGGTAAC R: CACATCATATGCGGCTCTG P: FAM-TGTTTGATTTGGCCTGGGCTCTTCG-BHQ1	111	Baek et al. (2023) [14]
CDV	P	F: CTGTCRGTAATCGAGRATTCGA R: GCCGAAAGAATATCCCCAGTTAG P: FAM-ATCTTCGCCAGARTCYTCAGTGCT-BHQ1	116	Halecker et al. (2021) [16]
FeLV	LTR	F: AACAGCAGAAGTTTCAAGGCC R. TTATAGCAGAAAGCGCGCG P: FAM-CCAGCAGTCTCCAGGCTCCCCA-BHQ1	131	Tandon et al. (2005) [17]

^a^ Primers and probes for real-time quantitative polymerase chain reaction (qPCR) to detect viral protein 2 (VP2) gene of feline parvovirus (FPV) and real-time quantitative reverse-transcription polymerase chain reaction (qRT-PCR) assays to detect 7b gene of feline coronavirus (FCoV), p30 gene of feline calicivirus (FCV), phosphoprotein (P) gene of canine distemper virus (CDV), and long terminal repeat (LTR) gene of feline leukemia virus (FeLV), adopted from previously described protocols. FAM, 6-carboxyfluorescein; BHQ1, Black Hole Quencher 1.

**Table 2:** Screening of viral pathogens using fecal samples collected from five Siberian tigers (*Panthera tigris altaica*) at Seoul Zoo.

No.	Name ^a^	Age (year)	Clinical Signs	WBC (10^3^/µL)	Virus Detection by Molecular Assays ^b^				
					FPV	FCoV	FCV	CDV	FeLV
1	Penza	13	Mild	NT	+	−	−	−	−
2	Haerang	1	Severe	4.89	+	−	−	−	−
3	Sarang	1	Severe	3.08	+	−	−	−	−
4	Parang ^c^	1	Death	0.66	NT	NT	NT	NT	NT
5	Miho	10	Healthy	NT	−	−	−	−	−
6	Kumkang	5	Healthy	NT	−	−	−	−	−

^a^ Four tigers including three sibling cubs (Haerang, Sarang, and Parang) and their mother (Penza) housed in the same breeding house were parvovirus (FPV)-positive, but the remaining two tigers (Miho and Kumkang) housed in a different nearby breeding building were not. ^b^ Results of qPCR assay for feline parvovirus (FPV) and qRT-PCR assays for feline coronavirus (FCoV), feline calicivirus (FCV), canine distemper virus (CDV), and feline leukemia virus (FeLV) were presented as positive (+) or negative (−). ^c^ The tiger was FPV-positive when tested using a commercial immunochromatography test kit but not when tested using molecular assays due to the lack of the submitted sample.

**Table 3:** Homology of VP2 nucleotide and amino acid sequences among the KTPV-2305, CPV-2, and FPV reference strains, as well as Korean FPV strains.

Virus Type		Strain	GenBank Number	Host (Year)	Homology of VP2 Gene with the KTPV-2305 Strain (%)	
					Nucleotide	Amino Acid
CPV-2 Reference strains	CPV-2	Pfizer/vaccine/06	EU914139	Dog	98.6	97.8
	CPV-2	Intervet/vaccine/06	FJ011098	Dog	98.6	97.9
	CPV-2a	PVFCAPC	M24003	Dog	98.9	98.1
	CPV-2b	PVCVP1VP2A/39	M74849	Dog	98.8	98.1
	CPV-2c	67/06	FJ005214	Dog	98.6	97.9
FPV Reference strains	FPV	Philips Roxane	M24002	Cat	99.5	99.5
	FPV	Purevax	EU498680	Cat	99.5	99.5
	FPV	Felocell	EU498681	Cat	99.5	99.7
Korean FPV strains	FPV	KF001	EU252145	Cat (2007)	99.4	99.8
	FPV	KF002	EU252146	Cat (2007)	99.7	99.8
	FPV	KF003	EU252147	Cat (2007)	99.7	99.8
	FPV	K2	HQ184189	Cat (2008)	99.9	99.8
	**FPV**	**K3**	**HQ184190**	**Cat (2008)**	**99.9**	**100.0**
	**FPV**	**K4**	**HQ184191**	**Cat (2008)**	**100.0**	**100.0**
	FPV	K7	HQ184192	Cat (2008)	99.5	99.8
	FPV	K22	HQ184193	Cat (2008)	99.4	99.8
	FPV	K23	HQ184194	Cat (2008)	99.7	99.8
	FPV	K49	HQ184195	Cat (2008)	99.7	99.7
	FPV	K50	HQ184196	Cat (2008)	99.7	99.1
	FPV	KS2	HQ184204	Cat (2008)	99.4	99.3
	FPV	KS11	HQ184197	Cat (2008)	99.4	99.8
	FPV	KS18	HQ184198	Cat (2008)	99.4	99.1
	FPV	KS23	HQ184199	Cat (2008)	99.6	99.7
	FPV	KS42	HQ184200	Cat (2008)	99.8	99.8
	FPV	KS45	HQ184201	Cat (2008)	99.5	99.1
	FPV	KS47	HQ184202	Cat (2008)	99.6	99.5
	FPV	KS58	HQ184203	Cat (2008)	99.7	99.8
	FPV	Gigucheon	MN400978	Cat (2017)	99.3	99.1
	FPV	Jun	MN400979	Cat (2017)	99.0	99.0
	FPV	Rara	MN400980	Cat (2017)	98.2	95.9
	**FPV**	**17D01**	**OP153925**	**Cat (2017)**	**99.8**	**100.0**
	**FPV**	**17D02**	**OP153926**	**Cat (2017)**	**99.8**	**100.0**
	FPV	Fe-P2	MN683826	Cat (2017)	99.0	99.0
	FPV	18D01	OP153927	Cat (2018)	99.5	99.8
	FPV	18Q234-1	MW035310	Dog (2018)	99.5	99.8
	**FPV**	**19D01**	**OP153928**	**Cat (2019)**	**99.8**	**100.0**
	**FPV**	**19D02**	**OP153929**	**Cat (2019)**	**99.8**	**100.0**
	**FPV**	**19D03**	**OP153930**	**Cat (2019)**	**99.7**	**100.0**
	**FPV**	**19D04**	**OP153931**	**Cat (2019)**	**99.8**	**100.0**
	FPV	19D05	OP153932	Cat (2019)	98.5	99.8
	**FPV**	**19SP_CK-8**	**MW035309**	**Dog (2019)**	**99.8**	**100.0**

Words in bold indicate the closest FPV strains with 100% nucleotide and/or amino acid homology with the VP2 gene of the KTPV-2305 strain detected in this study.

**Table 4:** Amino acid variation in the VP2 protein of the KTPV-2305 strain relative to the FPV and CPV-2 reference strains, as well as Korean FPV strains.

Virus Type	Strain	Variation in Amino Acid Residues at Positions in the VP2 Protein												
		80	87	93	101	103	232	300	305	323	426	562	564	568
In this study	KTPV-2305	K	M	K	**T**	V	**V**	A	D	D	N	**V**	N	A
FPV reference strains	FPV Philips Roxane	K	M	K	**I**	V	**I**	A	D	D	N	**L**	N	A
	FPV Purevax	K	M	K	**I**	V	**I**	A	D	D	N	**L**	N	A
	FPV Felocell	K	M	K	**T**	V	**I**	A	D	D	N	**L**	N	A
CPV-2 reference strains	CPV-2|Pfizer/vaccine/06	R	M	N	**I**	A	**I**	A	D	N	N	**V**	S	G
	CPV-2|Intervet/vaccine/06	R	M	N	**I**	A	**I**	A	D	N	N	**V**	S	G
	CPV-2a|PVFCAPC	R	L	N	**T**	A	**I**	G	Y	N	N	**V**	S	G
	CPV-2b|PVCVP1VP2A/39	R	L	N	**T**	A	**I**	G	Y	N	D	**V**	S	G
	CPV-2c|67/06	R	L	N	**T**	A	**I**	G	Y	N	E	**V**	S	G
Korean FPV strains	KF001	K	M	K	**T**	V	**V**	A	D	D	N	**V**	N	A
	KF002	K	M	K	**T**	V	**V**	A	D	D	N	**V**	N	A
	KF003	K	M	K	**T**	V	**V**	A	D	D	N	**V**	N	A
	K2	K	M	K	**T**	V	**V**	A	D	D	N	**V**	N	A
	K3	K	M	K	**T**	V	**V**	A	D	D	N	**V**	N	A
	K4	K	M	K	**T**	V	**V**	A	D	D	N	**V**	N	A
	K7	K	M	K	**T**	V	**V**	A	D	D	N	**V**	N	A
	K22	K	M	K	**T**	V	**V**	A	D	D	N	**V**	N	A
	K23	K	M	K	**T**	V	**V**	A	D	D	N	**V**	N	A
	K49	K	M	K	**T**	V	**V**	A	D	D	N	**V**	N	A
	K50	Q	M	K	**T**	V	**V**	A	D	D	N	**V**	N	A
	KS2	K	M	K	**T**	V	**I**	A	D	D	N	**V**	N	A
	KS11	K	M	K	**T**	V	**V**	A	D	D	N	**V**	N	A
	KS18	K	M	K	**T**	V	**V**	A	D	D	N	**V**	N	A
	KS23	K	M	K	**T**	V	**V**	A	D	D	N	**V**	N	A
	KS42	K	M	K	**T**	V	**V**	A	D	D	N	**V**	N	A
	KS45	K	M	K	**T**	V	**V**	A	N	D	N	**V**	N	A
	KS47	K	M	K	**T**	V	**I**	A	D	D	N	**V**	N	A
	KS58	K	M	K	**T**	V	**V**	A	D	D	N	**V**	N	A
	Gigucheon	K	M	K	**T**	V	**V**	A	D	D	N	**V**	N	A
	Jun	K	M	K	**T**	V	**V**	A	D	D	N	**V**	N	A
	Rara	K	M	K	**T**	V	**V**	A	D	D	N	**V**	N	A
	17D01	K	M	K	**T**	V	**V**	A	D	D	N	**V**	N	A
	17D02	K	M	K	**T**	V	**V**	A	D	D	N	**V**	N	A
	Fe-P2	K	M	K	**T**	V	**V**	A	D	D	N	**V**	N	A
	18D01	K	M	K	**T**	V	**V**	A	D	D	N	**V**	N	A
	18Q234-1	K	M	K	**T**	V	**V**	A	D	D	N	**V**	N	A
	19D01	K	M	K	**T**	V	**V**	A	D	D	N	**V**	N	A
	19D02	K	M	K	**T**	V	**V**	A	D	D	N	**V**	N	A
	19D03	K	M	K	**T**	V	**V**	A	D	D	N	**V**	N	A
	19D04	K	M	K	**T**	V	**V**	A	D	D	N	**V**	N	A
	19D05	K	M	K	**T**	V	**V**	A	D	D	N	**L**	N	A
	19SP_CK-8	K	M	K	**T**	V	**V**	A	D	D	N	**V**	N	A

A, alanine; D, aspartic acid; E, glutamic acid; G, glycine; I, isoleucine; K, lysine; L, leucine; M, methionine; N, asparagine; Q, Glutamine; R, arginine; S, Serine; T, threonine; V, valine; and Y, Tyrosine.

## Data Availability

The VP2 sequences of the virus obtained in this study were submitted to the GenBank database (accession number: OR365078).

## Supplementary Materials

The following supporting information can be downloaded at: https://www.mdpi.com/article/10.3390/ani13182991/s1, Table S1: Information on 578 global FPV sequences collected from GenBank database and amino acid residues at the position of 101, 232 and 562 in the VP2 sequences.
