# Supplementary material for: Epidemiological and Molecular Approaches for a Fatal Feline Panleukopenia Virus Infection of Captive Siberian Tigers (Panthera tigris altaica) in the Republic of Korea
Source: Animals (Basel). 2023 Sep 21;13(18):2991. doi: 10.3390/ani13182991 (PMC10526094; doi:10.3390/ani13182991)
Supplement: Supplementary file 1 [file animals-13-02991-s001.zip › animals-2586688-supplementary.pdf]

Table S1. Information on 578 global FPV sequences collected from GenBank database and amino acid residues at the position of 101, 232 and 562 in the VP2 sequences.

No.	Host	Year	Country	Strain	GenBank accession number	Amino acid residue of VP2 gene		
						101	232	562
1	Cat	1964	USA	4.us_64	EU659112	T	V	V
2	Cat	1967	USA	3.us_67	EU659111	T	V	V
3	Cat	1989	USA	Philips_Roxane	PVFCAPA	I	I	L
4	Cat	1989	USA	FPV-b_CU4	PVFCAPB	T	I	V
5	Cat	1993	Germany	FPV-377	FPU22188	T	V	V
6	Cat	1995	Japan	TU10	D78584	T	V	V
7	Cat	1995	USA	FPV-23	FPU22187	T	I	V
8	Cat	1995	USA	FPV-d	FPU22189	T	I	L
9	Cat	1996	Japan	Fukagawa	AB000054	I	V	V
10	Cat	1996	Japan	TU8	AB000070	I	I	V
11	Cat	1996	Japan	FPV-483	D88286	T	V	V
12	Cat	1996	Japan	Som4	AB000061	T	V	V
13	Cat	1996	Japan	94-1	AB000050	T	V	V
14	Cat	1996	Japan	Som1	AB000059	T	V	V
15	Cat	1996	Japan	PLI-IV	D88287	T	I	L
16	Cat	1996	Japan	Obihiro	AB000056	T	V	V
17	Cat	1996	Japan	AO1	AB000052	T	V	V
18	Cat	1996	Japan	TU4	AB000068	T	V	V
19	Cat	1996	Japan	TU2	AB000066	T	V	V
20	Cat	1996	Japan	TU12	AB000064	T	V	V
21	Cat	2001	Italy	198/01	EU498682	T	V	V
22	Cat	2001	Japan	V211	AB054227	T	V	V
23	Cat	2001	Japan	V142	AB054225	T	V	V
24	Cat	2001	Japan	V208	AB054226	T	V	V
25	Cat	2002	Italy	103/02	EU498684	T	V	V
26	Cat	2003	Italy	150/03	EU498685	T	V	V
27	Cat	2003	Italy	189/03	EU498686	T	V	V
28	Cat	2003	Italy	300/03	EU498687	T	V	V
29	Cat	2004	Italy	134/04-5	EU498691	T	V	V
30	Cat	2004	Italy	134/04-2	EU498689	T	V	V
31	Cat	2004	Italy	134/04-3	EU498690	T	V	V
32	Cat	2004	Italy	143/04	EU498692	T	V	V
33	Cat	2004	Italy	134/04-1	EU498688	T	V	V
34	Cat	2004	Italy	355/04	EU498693	T	V	V
35	Cat	2005	Italy	20/05	EU498694	T	V	V
36	Cat	2005	Italy	119/05	EU498695	T	V	V
37	Cat	2005	Portugal	46912/PT05	EU221279	T	V	V
38	Cat	2005	Portugal	52171/PT05	EU221278	T	V	V
39	Cat	2006	Italy	22/06	EU498696	T	V	V
40	Cat	2006	Italy	42/06-G6	EU498702	T	V	V
41	Cat	2006	Italy	42/06-G14	EU498708	T	V	V
42	Cat	2006	Italy	42/06-G4	EU498700	T	V	V
43	Cat	2006	Italy	228/06	EU498715	T	V	V
44	Cat	2006	Italy	42/06-G19	EU498712	T	V	V
45	Cat	2006	Italy	42/06-G1	EU498697	T	V	V
46	Cat	2006	Italy	42/06-17	EU498710	T	V	V
47	Cat	2006	Italy	42/06-G7	EU498703	T	V	V
48	Cat	2006	Italy	42/06-G10	EU498705	T	V	V
49	Cat	2006	Italy	42/06-G8	EU498704	T	V	V

50	Cat	2006	Italy	42/06-G2	EU498698	T	V	V
51	Cat	2006	Italy	42/06-G11	EU498706	T	V	V
52	Cat	2006	Italy	42/06-G3	EU498699	T	V	V
53	Cat	2006	Italy	42/06-18	EU498711	T	V	V
54	Cat	2006	Italy	42/06-12	EU498707	T	V	V
55	Cat	2006	Italy	42/06-G5	EU498701	T	V	V
56	Cat	2006	Italy	42/06-G16	EU498709	T	V	V
57	Cat	2006	Portugal	39897/PT06	EU221280	T	V	V
58	Cat	2006	Portugal	PT020/06	KT240128	T	V	V
59	Cat	2006	Portugal	PT006/06	KU248461	T	V	V
60	Cat	2006	Portugal	PT004/06	KU248459	T	V	V
61	Cat	2006	Portugal	PT005/06	KU248460	T	V	V
62	Cat	2006	Portugal	PT001/06	KU248456	T	V	V
63	Cat	2006	Portugal	PT003/06	KU248458	T	V	V
64	Cat	2006	Portugal	PT002/06	KU248457	T	V	V
65	Cat	2006	Portugal	31609/PT06	EU221281	T	V	V
66	Cat	2006	UK	97/06-11	EU498714	T	I	V
67	Cat	2006	UK	97/06-10	EU498713	T	V	V
68	Cat	2006	USA	kai.us.06	EU659115	T	V	V
69	Cat	2007	Argentina	FPV_ARG08	FJ440714	T	I	V
70	Cat	2007	Argentina	FPV_ARG05	FJ440711	T	I	V
71	Cat	2007	Argentina	FPV_ARG06	FJ440712	T	I	V
72	Cat	2007	Argentina	FPV_ARG07	FJ440713	T	I	V
73	Cat	2007	China	XJ-1	EF988660	T	V	V
74	Cat	2007	Hungary	1335/07	EU360959	T	V	V
75	Cat	2007	Hungary	933/07	EU360958	T	V	V
76	Cat	2007	Italy	443/07	EU498718	T	V	V
77	Cat	2007	Italy	498/07	EU498720	T	V	V
78	Cat	2007	Korea	KF002	EU252146	T	V	V
79	Cat	2007	Korea	KF003	EU252147	T	V	V
80	Cat	2007	Korea	KF001	EU252145	T	V	V
81	Cat	2007	Portugal	PT001/07	KT240129	T	V	V
82	Cat	2007	Portugal	PT002/07	KU248462	T	V	V
83	Cat	2007	UK	50/07-2	EU498717	I	I	L
84	Cat	2007	UK	490/07	EU498719	T	I	L
85	Cat	2007	UK	50/07-1	EU498716	T	I	V
86	Cat	2008	China	ChangC2007	FJ936171	T	I	V
87	Cat	2008	Italy	Purevax	EU498680	I	I	L
88	Cat	2008	Italy	41/02	EU498683	T	V	V
89	Cat	2008	Italy	Felocell	EU498681	T	I	L
90	Cat	2008	Korea	K3	HQ184190	T	V	V
91	Cat	2008	Korea	K4	HQ184191	T	V	V
92	Cat	2008	Korea	K49	HQ184195	T	V	V
93	Cat	2008	Korea	KS11	HQ184197	T	V	V
94	Cat	2008	Korea	K7	HQ184192	T	V	V
95	Cat	2008	Korea	K22	HQ184193	T	V	V
96	Cat	2008	Korea	K23	HQ184194	T	V	V
97	Cat	2008	Korea	KS58	HQ184203	T	V	V
98	Cat	2008	Korea	KS42	HQ184200	T	V	V
99	Cat	2008	Korea	K2	HQ184189	T	V	V
100	Cat	2008	Korea	KS47	HQ184202	T	I	V
101	Cat	2008	Korea	KS2	HQ184204	T	I	V
102	Cat	2008	Korea	KS23	HQ184199	T	V	V
103	Cat	2008	Korea	K50	HQ184196	T	V	V
104	Cat	2008	Korea	KS45	HQ184201	T	V	V
105	Cat	2008	Korea	KS18	HQ184198	T	V	V

106	Cat	2008	Portugal	PT015/08	KU248463	T	V	V
107	Cat	2008	Portugal	PT005/08	KT240130	T	V	V
108	Cat	2008	Portugal	PT022/08	KT240131	T	V	V
109	Cat	2011	Taiwan	FPV-1	JX048608	T	V	V
110	Cat	2012	China	FPV-GD(12/09/YGP)	KC473946	T	V	V
111	Cat	2012	Portugal	PT183/12	KT240132	T	V	V
112	Cat	2013	Belgium	MG132167A	KP769859	T	V	V
113	Cat	2013	Portugal	PT083/13	KT240133	T	V	V
114	Cat	2013	Portugal	PT210/13	KT240134	T	V	V
115	Cat	2013	USA	ND/979/2013	KJ813893	T	V	V
116	Cat	2014	China	HRB-CS1	KP280068	T	V	V
117	Cat	2014	China	FPV-BJ05	MH165482	T	I	L
118	Cat	2014	Portugal	PT264/14	KT240135	T	V	V
119	Cat	2014	Portugal	PT265/14	KU248464	T	V	V
120	Cat	2014	Portugal	PT271/14	KT240136	T	V	V
121	Cat	2015	China	FPV-BJ04	MH165481	T	I	L
122	Cat	2015	Italy	FPV_IZSSI_3201_1_15	KX434461	T	V	V
123	Cat	2015	Italy	FPV_IZSSI_42807_15	KX434462	T	I	V
124	Cat	2016	China	JL-04/16	MF541125	T	V	V
125	Cat	2016	China	16JZ0601	MK671154	T	V	V
126	Cat	2016	China	BJ-03/16	MF541120	T	V	V
127	Cat	2016	China	CC-02/16	MF541121	T	V	V
128	Cat	2016	China	GX01	MG924893	T	V	V
129	Cat	2016	China	16CC0806	MK671150	T	V	V
130	Cat	2016	China	HRB-01/16	MF541122	T	V	V
131	Cat	2016	China	16CC1106	MK671151	T	V	V
132	Cat	2016	China	16JL1205	MK671153	T	V	V
133	Cat	2016	China	F2016019	MH329286	T	V	V
134	Cat	2016	China	BC-02/16	MF541119	T	V	V
135	Cat	2016	China	16SY0601	MK671155	T	V	V
136	Cat	2016	China	16JL0804	MK671152	T	V	V
137	Cat	2016	China	16SY0711	MK671156	T	V	V
138	Cat	2016	China	Chengdu-03/2017	MK266790	T	V	V
139	Cat	2017	China	JL-01/17-03	MF541123	T	V	V
140	Cat	2017	China	17SP0503	MK671167	T	V	V
141	Cat	2017	China	JL-10/17-06	MF541128	T	V	V
142	Cat	2017	China	Beijing-L3/2018	MK266795	T	V	V
143	Cat	2017	China	17JLSY0701	MK671165	T	V	V
144	Cat	2017	China	JL-07/17-05	MF541127	T	V	V
145	Cat	2017	China	Shenyang-5/2017	MK266785	T	V	V
146	Cat	2017	China	Haerbin-13/2017	MK266784	T	V	V
147	Cat	2017	China	JT-01/17-03	MF541139	T	V	V
148	Cat	2017	China	Haerbin-05/2017	MK266782	T	V	V
149	Cat	2017	China	JL-33/17-05	MF541135	T	V	V
150	Cat	2017	China	17SY0302	MK671168	T	V	V
151	Cat	2017	China	17JLSY0901	MK671166	T	V	V
152	Cat	2017	China	JL-12/17-05	MF541129	T	V	V
153	Cat	2017	China	SMU-D4/2017	MZ442309	T	V	V
154	Cat	2017	China	Tianjin-02/2018	MK266794	T	V	V
155	Cat	2017	China	17DD0501	MK671160	T	V	V
156	Cat	2017	China	JL-34/17-05	MF541136	T	V	V
157	Cat	2017	China	JL-40/17-05	MF541137	T	V	V
158	Cat	2017	China	JL-03/17-05	MF541124	T	V	V
159	Cat	2017	China	17HRB1001	MK671163	T	V	V
160	Cat	2017	China	17BC0801	MK671158	T	V	V
161	Cat	2017	China	JL-20/17-05	MF541131	T	V	V

162	Cat	2017	China	JL-29/17-05	MF541134	T	V	V
163	Cat	2017	China	JL-19/17-06	MF541130	T	V	V
164	Cat	2017	China	17BC0704	MK671157	T	V	V
165	Cat	2017	China	17HRB0505	MK671162	T	V	V
166	Cat	2017	China	JL-47/17-05	MF541138	T	V	V
167	Cat	2017	China	Shenyang-19/2017	MK266786	T	V	V
168	Cat	2017	China	ZJFPV7	MW495833	T	V	V
169	Cat	2017	China	JL-3	MK295775	T	V	V
170	Cat	2017	China	Guiyang01/2017	MK266788	T	V	V
171	Cat	2017	China	17SY0503	MK671170	T	V	V
172	Cat	2017	China	17SY0902	MK671171	T	V	V
173	Cat	2017	China	Shenyang-01/2017	MK266792	T	V	V
174	Cat	2017	China	17CC0308	MK671159	T	V	V
175	Cat	2017	China	17SY0402	MK671169	T	V	V
176	Cat	2017	China	17JL0704	MK671164	T	V	V
177	Cat	2017	China	SMU-D3/2017	MZ442312	T	V	V
178	Cat	2017	China	SP-01/16	MF541140	T	V	V
179	Cat	2017	China	JL-24/17-05	MF541132	T	V	V
180	Cat	2017	China	JL-28/17-05	MF541133	T	V	V
181	Cat	2017	China	JL-04/17-03	MF541126	T	V	V
182	Cat	2017	China	ZJFPV5	MW495831	T	V	V
183	Cat	2017	China	Beijing-01/2018	MK266797	T	V	V
184	Cat	2017	China	17DD0902	MK671161	T	V	V
185	Cat	2017	China	Chengdu-01/2017	MK266791	T	V	V
186	Cat	2017	China	ZJFPV13	MW495838	T	V	V
187	Cat	2017	Korea	Fe-P2	MN683826	T	V	V
188	Cat	2017	Korea	17D02	OP153926	T	V	V
189	Cat	2017	Korea	17D01	OP153925	T	V	V
190	Cat	2017	Korea	Gigucheon	MN400978	T	V	V
191	Cat	2017	Korea	Jun	MN400979	T	V	V
192	Cat	2017	Korea	Rara	MN400980	T	V	V
193	Cat	2018	Canada	K1830	OM640096	T	I	L
194	Cat	2018	China	18HRB0801	MK671178	T	V	V
195	Cat	2018	China	Shenyang-05/2017	MK266798	T	V	V
196	Cat	2018	China	DL06-2018	ON646206	T	V	V
197	Cat	2018	China	18QQHE0503	MK671186	T	V	V
198	Cat	2018	China	18DD0302	MK671176	T	V	V
199	Cat	2018	China	18HRB0102	MK671177	T	V	V
200	Cat	2018	China	18CC0718	MK671174	T	V	V
201	Cat	2018	China	Haerbin-01/2018	MK266783	T	V	V
202	Cat	2018	China	SMU-D53/2018	MZ442305	T	V	V
203	Cat	2018	China	DLC05	MN419001	T	V	V
204	Cat	2018	China	DLC06	MN419002	T	V	V
205	Cat	2018	China	ZJFPV10	MW495835	T	V	V
206	Cat	2018	China	DL03-2018	ON646203	T	V	V
207	Cat	2018	China	18SY0102	MK671188	T	V	V
208	Cat	2018	China	18JZ0501	MK671182	T	V	V
209	Cat	2018	China	Shenyang-41/2017	MK266787	T	V	V
210	Cat	2018	China	DLC01	MN418997	T	V	V
211	Cat	2018	China	18CC0102	MK671173	T	V	V
212	Cat	2018	China	DLC02	MN418998	T	V	V
213	Cat	2018	China	SMU-D28/2018	MZ442311	T	V	V
214	Cat	2018	China	SMU-D50/2018	MZ442306	T	V	V
215	Cat	2018	China	18LY0701	MK671183	T	V	V
216	Cat	2018	China	Guiyang01/2017	MK266789	T	V	V
217	Cat	2018	China	18HRB1002	MK671179	T	V	V

218	Cat	2018	China	18LY0902	MK671185	T	V	V
219	Cat	2018	China	18LY0801	MK671184	T	V	V
220	Cat	2018	China	18CC0909	MK671175	T	V	V
221	Cat	2018	China	ZJFPV2	MW495829	T	V	V
222	Cat	2018	China	ZJFPV15	MW495840	T	V	V
223	Cat	2018	China	DL05-2018	ON646205	T	V	V
224	Cat	2018	China	Tanjin-01/2018	MK266793	T	V	V
225	Cat	2018	China	DL07-2018	ON646207	T	V	V
226	Cat	2018	China	DLC04	MN419000	T	V	V
227	Cat	2018	China	ZJFPV4	MW495830	T	V	V
228	Cat	2018	China	DLC03	MN418999	T	V	V
229	Cat	2018	China	DL01-2018	ON646201	T	V	V
230	Cat	2018	China	DL02-2018	ON646202	T	V	V
231	Cat	2018	China	18JL0602	MK671181	T	V	V
232	Cat	2018	China	18BC0504	MK671172	T	V	V
233	Cat	2018	China	18JL0105	MK671180	T	V	V
234	Cat	2018	China	18SP0701	MK671187	T	V	V
235	Cat	2018	China	Beijing-L4/2018	MK266796	T	V	V
236	Cat	2018	China	ZJFPV8	MW495834	T	V	V
237	Cat	2018	China	ZJFPV14	MW495839	T	V	V
238	Cat	2018	China	SMU-D46/2018	MZ442307	T	V	V
239	Cat	2018	China	Jilin5/2017	MK266799	T	V	V
240	Cat	2018	India	TN/FPV/2018	MH559110	T	V	V
241	Cat	2018	Korea	18D01	OP153927	T	V	V
242	Cat	2018	Thailand	CMU-F12	MK425504	T	V	V
243	Cat	2018	Thailand	CMU-F34	MK425507	T	V	V
244	Cat	2018	Thailand	CMU-F04	MK425500	T	V	V
245	Cat	2018	Thailand	CMU-F13	MK425505	T	V	V
246	Cat	2018	Thailand	CMU-F10	MK425503	T	I	V
247	Cat	2018	Thailand	18R217C/TH/2018	MN127779	T	V	V
248	Cat	2018	Thailand	CMU-F14	MK425506	T	V	V
249	Cat	2018	Thailand	CMU-F09	MK425502	T	V	V
250	Cat	2018	Thailand	CMU-F01	MK425497	T	V	V
251	Cat	2018	Thailand	CMU-F03	MK425499	T	V	V
252	Cat	2018	Thailand	CMU-F02	MK425498	T	V	V
253	Cat	2018	Thailand	CMU-F07	MK425501	T	V	V
254	Cat	2018	Vietnam	F3	MT857285	T	V	V
255	Cat	2018	Vietnam	F8	MT857271	T	V	V
256	Cat	2018	Vietnam	F4	MT857286	T	V	V
257	Cat	2018	Vietnam	F6	MT857269	T	V	V
258	Cat	2018	Vietnam	F1	MT857283	T	V	V
259	Cat	2018	Vietnam	F2	MT857284	T	V	V
260	Cat	2018	Vietnam	F5	MT857268	T	V	V
261	Cat	2018	Vietnam	F7	MT857270	T	V	V
262	Cat	2018	Vietnam	F9	MT857272	T	V	V
263	Cat	2019	China	BJ061	MT270578	T	V	V
264	Cat	2019	China	BJ577	MT270545	T	V	V
265	Cat	2019	China	BJ277	MT270570	T	V	V
266	Cat	2019	China	BJ318	MT270567	T	V	V
267	Cat	2019	China	DL17-2019	ON646215	T	V	V
268	Cat	2019	China	BJ663	MT270534	T	V	V
269	Cat	2019	China	BJ379	MT270563	T	V	V
270	Cat	2019	China	BJ319	MT270566	T	V	V
271	Cat	2019	China	BJ372	MT270564	T	V	V
272	Cat	2019	China	BJ006	MT270585	T	V	V
273	Cat	2019	China	BJ588	MT270543	T	V	V

274	Cat	2019	China	BJ020	MT270583	T	V	V
275	Cat	2019	China	BJ016	MT270584	T	V	V
276	Cat	2019	China	DL12-2019	ON646210	T	V	V
277	Cat	2019	China	HBSJZ19-02	OP471919	T	V	V
278	Cat	2019	China	DLC14	MN419011	T	V	V
279	Cat	2019	China	BJ078	MT270577	T	V	V
280	Cat	2019	China	BJ562	MT270549	T	V	V
281	Cat	2019	China	BJ574	MT270546	T	V	V
282	Cat	2019	China	DLC10	MN419007	T	V	V
283	Cat	2019	China	DLC16	MN419013	T	V	V
284	Cat	2019	China	BJ025	MT270582	T	V	V
285	Cat	2019	China	DLC92	MN419006	T	V	V
286	Cat	2019	China	DLC13	MN419010	T	V	V
287	Cat	2019	China	DLC08	MN419004	T	V	V
288	Cat	2019	China	DLC07	MN419003	T	V	V
289	Cat	2019	China	SMU-D74/2019	MZ442304	T	V	V
290	Cat	2019	China	SMU-D87/2019	MZ442303	T	V	V
291	Cat	2019	China	DLC11	MN419008	T	V	V
292	Cat	2019	China	DL20-2019	ON646218	T	I	V
293	Cat	2019	China	BJ629	MT270538	T	V	V
294	Cat	2019	China	BJ638	MT270537	T	V	V
295	Cat	2019	China	BJ624	MT270540	T	V	V
296	Cat	2019	China	BJ435	MT270559	T	V	V
297	Cat	2019	China	BJ338	MT270565	T	V	V
298	Cat	2019	China	BJ128	MT270574	T	V	V
299	Cat	2019	China	BJ540	MT270553	T	V	V
300	Cat	2019	China	BJ416	MT270560	T	V	V
301	Cat	2019	China	ZJFPV16	MW495841	T	V	V
302	Cat	2019	China	BJ619	MT270541	T	V	V
303	Cat	2019	China	BJ133	MT270573	T	V	V
304	Cat	2019	China	BJ572	MT270547	T	V	V
305	Cat	2019	China	BJ309	MT270568	T	V	V
306	Cat	2019	China	BJ481	MT270556	T	V	V
307	Cat	2019	China	ZJFPV11	MW495836	T	V	V
308	Cat	2019	China	DLC15	MN419012	T	V	V
309	Cat	2019	China	ZJFPV6	MW495832	T	V	V
310	Cat	2019	China	BJ625	MT270539	T	V	V
311	Cat	2019	China	BJ094	MT270575	T	V	V
312	Cat	2019	China	HF1	MT614366	T	V	V
313	Cat	2019	China	BJ700	MT270532	T	V	V
314	Cat	2019	China	BJ050	MT270581	T	V	V
315	Cat	2019	China	BJ565	MT270548	T	V	V
316	Cat	2019	China	BJ557	MT270550	T	V	V
317	Cat	2019	China	CC19-02	OP471917	T	V	V
318	Cat	2019	China	BJ501	MT270555	T	V	V
319	Cat	2019	China	JN-FPV-11	MZ836347	T	V	V
320	Cat	2019	China	HBSJZ19-01	OP471918	T	V	V
321	Cat	2019	China	F-D33	OL547736	T	V	V
322	Cat	2019	China	BJ058	MT270579	T	V	V
323	Cat	2019	China	BJ698	MT270533	T	V	V
324	Cat	2019	China	BJ405	MT270561	T	V	V
325	Cat	2019	China	BJ440	MT270558	T	V	V
326	Cat	2019	China	BJ396	MT270562	T	V	V
327	Cat	2019	China	BJ235	MT270572	T	V	V
328	Cat	2019	China	BJ502	MT270554	T	V	V
329	Cat	2019	China	BJ552	MT270552	T	V	V

330	Cat	2019	China	BJ644	MT270536	T	V	V
331	Cat	2019	China	BJ554	MT270551	T	V	V
332	Cat	2019	China	BJ662	MT270535	T	V	V
333	Cat	2019	China	BJ461	MT270557	T	V	V
334	Cat	2019	China	F-D34	OL547735	T	V	V
335	Cat	2019	China	BJ051	MT270580	T	V	V
336	Cat	2019	China	BJ308	MT270569	T	V	V
337	Cat	2019	China	BJ582	MT270544	T	V	V
338	Cat	2019	China	BJ728	MT270531	T	V	V
339	Cat	2019	China	BJ594	MT270542	T	V	V
340	Cat	2019	China	F-D35	OL547733	T	V	V
341	Cat	2019	China	DL08-2019	ON646208	T	V	L
342	Cat	2019	China	DL09-2019	ON646209	T	V	V
343	Cat	2019	China	DL13-2019	ON646211	T	I	V
344	Cat	2019	China	DL18-2019	ON646216	T	V	V
345	Cat	2019	China	JN-FPV-12	MZ836378	T	V	V
346	Cat	2019	China	FPV-SD/2019/4	OK384312	T	V	V
347	Cat	2019	China	FPV-SD/2019/2	OK384310	T	V	V
348	Cat	2019	China	FPV-SD/2019/5	OK384313	T	V	V
349	Cat	2019	China	FPV-SD/2019/3	OK384311	T	V	V
350	Cat	2019	China	FPV-SD/2019/6	OK384314	T	V	V
351	Cat	2019	China	DL14-2019	ON646212	T	V	V
352	Cat	2019	China	DL16-2019	ON646214	T	V	V
353	Cat	2019	China	DLC12	MN419009	T	V	V
354	Cat	2019	China	BJ090	MT270576	T	V	V
355	Cat	2019	China	DLC91	MN419005	T	I	L
356	Cat	2019	China	DL19-2019	ON646217	T	I	L
357	Cat	2019	China	DL15-2019	ON646213	T	V	V
358	Cat	2019	China	ZJFPV12	MW495837	T	V	V
359	Cat	2019	China	BJ240	MT270571	T	V	V
360	Cat	2019	China	FPV-SX	MT892650	T	V	V
361	Cat	2019	Egypt	EGY/2019/39-566	OM937916	T	V	V
362	Cat	2019	India	CV3	MT078770	T	V	V
363	Cat	2019	India	BCC1	MT078767	T	V	V
364	Cat	2019	India	CPF6	MT078769	T	V	V
365	Cat	2019	India	PDC3	MT078771	T	V	V
366	Cat	2019	India	BCC13	MT078768	T	V	V
367	Cat	2019	Korea	19D02	OP153929	T	V	V
368	Cat	2019	Korea	19D01	OP153928	T	V	V
369	Cat	2019	Korea	19D04	OP153931	T	V	V
370	Cat	2019	Korea	19D03	OP153930	T	V	V
371	Cat	2019	Korea	19D05	OP153932	T	V	L
372	Cat	2019	Thailand	19R124C/TH/2019	MN127781	T	I	L
373	Cat	2019	Thailand	19R81C/TH/2019	MN127780	T	I	V
374	Cat	2019	UK	Cat_1	MW926314	T	I	L
375	Cat	2019	UK	Cat_3	MW926316	T	V	V
376	Cat	2019	Vietnam	F10	MT857273	T	V	V
377	Cat	2019	Vietnam	F11	MT857274	T	V	V
378	Cat	2019	Vietnam	F12	MT857275	T	V	V
379	Cat	2019	Vietnam	F13	MT857276	T	V	V
380	Cat	2019	Vietnam	F19	MT857282	T	V	V
381	Cat	2019	Vietnam	F14	MT857277	T	V	V
382	Cat	2019	Vietnam	F15	MT857278	T	V	V
383	Cat	2019	Vietnam	F18	MT857281	T	V	V
384	Cat	2019	Vietnam	F17	MT857280	T	V	V
385	Cat	2019	Vietnam	F16	MT857279	T	V	V

386	Cat	2020	China	ZJFPV23	MW495848	T	V	V
387	Cat	2020	China	SH-118	MW017625	T	V	V
388	Cat	2020	China	TZ-FPV-108	MZ836369	T	V	V
389	Cat	2020	China	TZ-FPV-99	MZ836371	T	V	V
390	Cat	2020	China	JN-FPV-92	MZ836374	T	V	V
391	Cat	2020	China	TZ-FPV-193	MZ836360	T	V	V
392	Cat	2020	China	JN-FPV-91	MZ836375	T	V	V
393	Cat	2020	China	TZ-FPV-195	MZ836359	T	V	V
394	Cat	2020	China	TZ-FPV-104	MZ836370	T	V	V
395	Cat	2020	China	JSYZ-122	MW017628	T	V	V
396	Cat	2020	China	TZ-FPV-122	MZ836366	T	V	V
397	Cat	2020	China	TZ-FPV-148	MZ836362	T	V	V
398	Cat	2020	China	JN-FPV-87	MZ836377	T	V	V
399	Cat	2020	China	JN-FPV-90	MZ836376	T	V	V
400	Cat	2020	China	TZ-FPV-124	MZ836365	T	V	V
401	Cat	2020	China	SH-121	MW017627	T	V	V
402	Cat	2020	China	TZ-FPV-242	MZ836351	T	V	V
403	Cat	2020	China	cf094par01-8	MZ357120	T	V	V
404	Cat	2020	China	F-A	OL547737	T	V	V
405	Cat	2020	China	ZJFPV20	MW495845	T	V	V
406	Cat	2020	China	FPV-SH2001	MW650831	T	V	V
407	Cat	2020	China	TZ-FPV-239	MZ836354	T	V	V
408	Cat	2020	China	JSYZ-168	MW791426	T	V	V
409	Cat	2020	China	ZJHN-126	MW017631	T	V	V
410	Cat	2020	China	SH-120	MW017626	T	V	V
411	Cat	2020	China	TZ-FPV-112	MZ836368	T	V	V
412	Cat	2020	China	SMU-F33/2020	MZ442302	T	V	V
413	Cat	2020	China	ZJFPV17	MW495842	T	V	V
414	Cat	2020	China	JSYZ-124	MW017630	T	V	V
415	Cat	2020	China	TZ-FPV-235	MZ836358	T	V	V
416	Cat	2020	China	JN-FPV-96	MZ836373	T	V	V
417	Cat	2020	China	SMU-SC20-6/2020	MZ442314	T	V	V
418	Cat	2020	China	F-D	OL547732	T	V	V
419	Cat	2020	China	TZ-FPV-133	MZ836364	T	V	V
420	Cat	2020	China	ZJFPV18	MW495843	T	V	V
421	Cat	2020	China	TZ-FPV-240	MZ836353	T	V	V
422	Cat	2020	China	FPV-SH2003	MW811187	T	V	V
423	Cat	2020	China	JSYZ-169	MW791427	T	V	V
424	Cat	2020	China	SMU-SC20-2/2020	MZ442313	T	V	V
425	Cat	2020	China	TZ-FPV-185	MZ836361	T	V	V
426	Cat	2020	China	ZJFPV19	MW495844	T	V	V
427	Cat	2020	China	FPV-SH2002	MW659466	T	V	V
428	Cat	2020	China	TZ-FPV-241	MZ836352	T	V	V
429	Cat	2020	China	TZ-FPV-238	MZ836355	T	V	V
430	Cat	2020	China	TZ-FPV-135	MZ836363	T	V	V
431	Cat	2020	China	JSYZ-123	MW017629	T	V	V
432	Cat	2020	China	TZ-FPV-237	MZ836356	T	V	V
433	Cat	2020	China	F-E	OL547734	T	V	V
434	Cat	2020	China	ZJFPV22	MW495847	T	V	V
435	Cat	2020	China	TZ-FPV-119	MZ836367	T	V	V
436	Cat	2020	China	ZJFPV21	MW495846	T	V	V
437	Cat	2020	China	TZ-FPV-243	MZ836350	T	V	V
438	Cat	2020	China	TZ-FPV-236	MZ836357	T	V	V
439	Cat	2020	Turkey	Barut	MZ391096	T	V	V
440	Cat	2020	Turkey	kucuk	MZ391097	T	V	V
441	Cat	2021	China	Yanji27	OM918773	T	V	V

442	Cat	2021	China	F-18	MZ913313	T	V	V
443	Cat	2021	China	Yanji4	OM885374	T	V	V
444	Cat	2021	China	Yanji25	OM918772	T	V	V
445	Cat	2021	China	Yanji26	OM918784	T	V	V
446	Cat	2021	China	Yanji10	OM885379	T	V	V
447	Cat	2021	China	Yanji33	OM918779	T	V	V
448	Cat	2021	China	Yanji31	OM918777	T	V	V
449	Cat	2021	China	Yanji	OM212011	T	V	V
450	Cat	2021	China	Yanji24	OM918771	T	V	V
451	Cat	2021	China	Yanji17	OM918783	T	V	V
452	Cat	2021	China	Yanji29	OM918775	T	V	V
453	Cat	2021	China	Yanji28	OM918774	T	V	V
454	Cat	2021	China	Yanji5	OM885375	T	V	V
455	Cat	2021	China	Yanji12	OM885381	T	V	V
456	Cat	2021	China	Yanji13	OM885382	T	V	V
457	Cat	2021	China	Yanji18	OM885384	T	V	V
458	Cat	2021	China	Yanji37	OM918785	T	V	V
459	Cat	2021	China	F-C	OL547731	T	V	V
460	Cat	2021	China	Yanji38	OM918782	T	V	V
461	Cat	2021	China	Yanji35	OM918780	T	V	V
462	Cat	2021	China	JSNJ-21G5	OP796709	T	V	V
463	Cat	2021	China	SH-21D2	OP796706	T	V	V
464	Cat	2021	China	JSNJ-21G4	OP796708	T	G	V
465	Cat	2021	China	SH-21D4	OP796707	T	V	V
466	Cat	2021	China	Yanji32	OM918778	T	V	V
467	Cat	2021	China	Yanji9	OM885378	T	V	V
468	Cat	2021	China	Yanji15	OM885383	T	V	V
469	Cat	2021	China	Yanji8	OM885377	T	V	V
470	Cat	2021	China	Yanji30	OM918776	T	V	V
471	Cat	2021	China	Yanji6	OM885376	T	V	V
472	Cat	2021	China	Yanji3	OM885373	T	V	V
473	Cat	2021	China	Yanji11	OM885380	T	V	V
474	Cat	2021	Egypt	EGY/2021/139-188	OM638043	T	V	V
475	Cat	2021	Italy	ITA/2021/164-1	OM638042	T	V	V
476	Cat	2022	China	ZJHN-2207	OP796715	T	V	V
477	Cat	2022	China	ZJHN-2206	OP796714	T	V	V
478	Cat	2022	China	ZJHZ-2204	OP796712	T	V	V
479	Cat	2022	China	ZJHN-2205	OP796713	T	V	V
480	Cat	2022	China	ZJHN-2208	OP796716	T	V	V
481	Cat	2022	China	ZJHZ-2203	OP796711	T	V	V
482	Cat	2022	China	ZJHZ-2202	OP796710	T	V	V
483	Cat	*	*	RCP_vaccine	ON605652	I	I	L
484	Dog	2017	Viet_Nam	HN40AA	MK357740	T	V	V
485	Dog	2017	Viet_Nam	HN3	MK357739	T	V	V
486	Dog	2017	Viet_Nam	HN39AA	MK357738	T	V	V
487	Dog	2018	Korea	18Q234-1	MW035310	T	V	V
488	Dog	2018	Viet_Nam	HN10	MK357741	T	V	V
489	Dog	2018	Viet_Nam	HN7	MK357743	T	V	V
490	Dog	2018	Viet_Nam	HN41AA	MK357742	T	V	V
491	Dog	2019	Australia	AP._Roseworthy/SA/5371/20	MZ362883	T	V	V
492	Dog	2019	China	C-1	MZ913317	T	V	V
493	Dog	2019	China	C-F9	MZ913315	T	V	V
494	Dog	2019	China	C-F88	MZ913316	T	V	V
495	Dog	2019	China	C-9	MZ913319	T	V	V
496	Dog	2019	China	C-DY6	OK128324	T	V	V
497	Dog	2019	China	JSYZ-85	MW017596	T	V	V

498	Dog	2019	China	C-4	MZ913318	T	V	V
499	Dog	2019	Korea	19SP_CK-8	MW035309	T	V	V
500	Dog	2020	China	ZJHN-135	MW017616	T	V	V
501	Dog	2020	China	ZJHN-138	MW017618	T	V	V
502	Dog	2021	China	D7	OK128325	T	V	V
503	Dog	2021	China	D13	MZ913314	T	V	V
504	Tiger	1999	China	FPV-G	MG764510	T	I	V
505	Tiger	2005	China	GT-2	AY955826	I	I	V
506	Tiger	2006	Portugal	Tiger/PT06	EF418568	T	V	V
507	Tiger	2007	China	HT-262	EU697383	T	V	V
508	Tiger	2007	China	HT-374	EU697386	T	V	V
509	Tiger	2007	China	HT-290	EU697384	T	V	V
510	Tiger	2007	China	HT-163	EU697387	T	V	V
511	Tiger	2008	China	FJ405225	FJ405225	T	V	V
512	Tiger	2016	China	HN-ZZ1	KX685354	T	V	V
513	Tiger	2017	China	CHJL-Siberian_Tiger-01/2017	MK982094	T	V	V
514	Tiger	2019	China	MHS2019	MN908257	T	V	V
515	Tiger	2023	Korea	KTPV-2305	OR365078	T	V	V
516	Raccoon	1978	USA	TX/Rac1.2/78	JN867596	T	I	V
517	Raccoon	1978	USA	TX/Rac3/1978	KM624023	T	I	V
518	Raccoon	1978	USA	TX/Rac2.2/78	JN867595	T	I	V
519	Raccoon	1990	USA	NJ/RPV-6/90	JN867594	T	V	V
520	Raccoon	2010	Canada	RC9/BC_2010	MF069446	T	V	V
521	Raccoon	2010	USA	CA/208-A/10	JN867593	T	V	V
522	Raccoon	2012	USA	MA/190/2012	KJ813894	T	V	V
523	Raccoon	2012	USA	MA/188/2012	KJ813895	T	V	V
524	Raccoon	2012	USA	GA/1/12	JX475270	T	I	L
525	Raccoon	2015	Canada	RC6/BC_2015	MF069445	T	V	V
526	Raccoon	2016	Canada	RC18/BC_2016	MF069447	T	V	V
527	Lion	1989	USA	8b.us_89	EU659114	I	V	V
528	Lion	1989	USA	8a.us_89	EU659113	T	V	V
529	Lion	2006	Portugal	Lion/PT06	EF418569	T	V	V
530	Lion	2015	China	FPV-L	MG764511	T	V	V
531	Lion	2020	China	HNZZ2	MZ005633	T	V	V
532	Giant panda	2018	China	gpfe267par01-12	MZ357122	T	V	V
533	Giant panda	2018	China	Giant_panda/CD/2018	MZ322607	T	V	V
534	Giant panda	2018	China	Giant_panda/CD/2018	MW091487	T	V	V
535	Giant panda	2018	China	Giant_panda/CD-2/2018	MW091486	T	V	V
536	Giant panda	2020	China	AMPV2020	MZ712026	T	V	V
537	Red panda	2020	China	rpfe013par01-5	MZ357119	T	V	V
538	Giant panda	2020	China	gpfe016par01-5	MW331496	T	V	V
539	American mink	2017	Canada	MIVI-21/BC_2017	MN862746	T	V	V
540	American mink	2018	Canada	MIVI-34/BC_2018	MN862743	T	V	V
541	American mink	2019	Canada	MIVI-72/BC_2019	MN862747	T	V	V
542	American pine marten	2016	Canada	MAVI-36/BC_2016	MN862744	T	V	V
543	American pine marten	2017	Canada	MAHG-3/BC_2017	MN862745	T	I	L
544	Arctic fox	1983	Finland	BFPV	MN451652	T	V	V
545	Arctic fox	1995	USA	BFPV-1,1983	FPU22185	T	V	V
546	Asian palm civet	2007	Hungary	389/07	EU145593	T	V	V
547	Badger	2019	Italy	245-1478_FPLV_BADGER_2019_ITALY	MT274378	T	V	V
548	Banded linsang	2015	Thailand	FPV-VT01	MH669800	T	V	V
549	Blue fox	2008	China	BFPV	GQ857595	I	I	L
550	Cheetah	2019	China	SY-01/2019	MT178243	T	V	V
551	Egyptian mongoose	2009	Portugal	PT09	JF422105	T	V	V
552	Jaguar	1986	China	HH-1/86	KX900570	T	V	V
553	Monkey	2008	China	BJ-22	FJ231389	I	I	V

554	Puma concolor	2010	USA	CO/977/10	JX475254	T	V	V
555	Puma concolor	2010	USA	CO/952/10	JX475245	T	V	V
556	Puma concolor	2010	USA	CO/545/10	JX475259	T	V	V
557	Puma concolor	2010	USA	CO/546/10	JX475253	T	I	L
558	Puma concolor	2011	USA	CO/1103/11	JX475256	T	V	V
559	Red fox	2017	Italy	51_FOX_2017_ITALY_SYLVATIC_FPLV	MT274377	T	V	V
560	River otter	2019	Canada	OTVI-16/BC_2019	MN862748	T	V	V
561	River otter	2019	Canada	OTVI-3/BC_2019	MN862749	T	V	V
562	*	1986	USA	pEH20	PVFVP	T	I	L
563	*	1990	Australia	193/70	X55115	I	V	V
564	*	1990	USA	CU-4	PVFPVC	I	V	V
565	*	1997	Taiwan	FPV-T1	AF015223	I	I	V
566	*	2005	China	JF-3	DQ099431	T	V	V
567	*	2005	China	ZF-5	DQ099430	T	V	V
568	*	2006	China	HT-69	DQ474235	T	V	V
569	*	2006	China	JF-1	DQ474236	T	V	V
570	*	2006	China	SM-4	DQ474238	T	I	V
571	*	2006	China	GT-3	DQ474237	T	V	V
572	*	2006	Japan	AB262659	AB262659	T	V	V
573	*	2007	Argentina	ARG01	EU018145	T	I	V
574	*	2007	Argentina	ARG04	EU018142	T	I	V
575	*	2007	Argentina	ARG02	EU018144	T	I	V
576	*	2007	Argentina	ARG03	EU018143	T	I	V
577	*	2018	China	FPLV-QDDX	MK301396	T	V	V
578	*	*	*	Gercules-Biocentr	AY665655	T	V	V

* It was not available in the GenBank data.
